# Efficacy and Safety of Antivirals in Lactating Women with Herpesviridae Infections: A Systematic Review

**DOI:** 10.3390/v17040538

**Published:** 2025-04-07

**Authors:** Vasiliki Kallia, Georgios Schinas, Georgios Karagiannopoulos, Karolina Akinosoglou

**Affiliations:** 1 Fourth of Internal Medicine, University General Hospital Attikon, Chaidari, 12462 Athens, Greece; vasilikikallia@gmail.com; 2 National and Kapodistrian University of Athens, Vasilissis Sofias 98, 11528 Athens, Greece; karayianno@gmail.com; 3 School of Medicine, University of Patras, 26504 Rio, Greece; akin@upatras.gr; 4 First Department of Internal Medicine, Laiko General Hospital, School of Medicine, National and Kapodistrian University of Athens, 11527 Athens, Greece; 5 Department of Internal Medicine and Division of Infectious Diseases, University General Hospital of Patras, 26504 Rio, Greece

**Keywords:** Herpesviridae infections, antiviral agents, lactation, breastfeeding, acyclovir, valacyclovir, Cytomegalovirus, varicella-zoster virus, Epstein–Barr virus, herpes simplex virus

## Abstract

Herpesviruses are prevalent pathogens affecting lactating women, yet the safety and efficacy of antiviral therapies in this population remain underexplored. This systematic review evaluates the safety and efficacy of antiviral therapies for Herpesviridae infections, including CMV, VZV, EBV, and HSV, in lactating mothers. A comprehensive literature search was conducted using PubMed, Cochrane Library, and Scopus, alongside specialized databases like LactMed. Twelve studies were included, comprising three randomized control trials, five observational studies, and four case reports. Quality assessment using Joanna Briggs Institute tools indicated moderate-to-high methodological quality for the trials and consistent strengths in case reports, though some limitations were noted. Results suggest that antiviral agents, particularly acyclovir and valacyclovir, are generally safe for breastfeeding mothers, with minimal infant exposure and low risk of adverse effects. However, the virologic benefits appear modest, and most studies focused on HIV co-infected populations, limiting generalizability to lactating women without HIV. In conclusion, while current evidence supports the use of specific antivirals during lactation, there is a critical need for further research to address existing knowledge gaps and optimize treatment strategies for both mothers and infants.

## 1. Introduction

Herpesviruses represent widespread pathogens affecting humans globally, with eight members commonly infecting humans [1]. The most significant human herpesviruses include herpes simplex virus (HSV) HSV-1, HSV-2, varicella-zoster virus (VZV), Epstein–Barr virus (EBV), Cytomegalovirus (CMV), and Human Herpes Virus (HHV) HHV-6A, HHV-6B, and HHV-7 [2]. Herpesviridae infections are common among pregnant women, with HSV affecting 2–3% of pregnancies [3]. CMV, VZV, EBV, and HSV can cause hepatitis during pregnancy, with CMV posing a high risk of birth defects [4]. While vertical transmission during pregnancy is rare (<1%), the risk increases significantly during labor for women with active lesions or asymptomatic viral shedding [3,4]. Neonatal HSV infection can have severe consequences, including central nervous system involvement and mortality [5]. Primary or first-episode genital herpes during pregnancy carries a higher transmission risk than recurrent infections [5]. Prevention, screening, and antiviral management of these infections are crucial for reducing maternal and neonatal morbidity, commonly during the postpartum period [4].

However, the assessment of antiviral therapies in lactating women remains a complex and underexplored area of clinical research. Conducting robust clinical trials to evaluate the safety and efficacy of antiviral agents in lactating mothers is fraught with ethical, scientific, and practical challenges. Ethical concerns stem from the potential risk of infant exposure through breast milk, making randomized controlled trials in this population challenging and often ethically untenable [6]. Additionally, translating findings from animal models to human outcomes is problematic due to physiological differences in drug metabolism and excretion, resulting in critical knowledge gaps concerning the safety profiles of antivirals for breastfed infants [7]. These methodological and logistical barriers are further compounded by recruitment and participation difficulties. Many lactating women may be hesitant to enroll in studies that could potentially disrupt breastfeeding or pose even a perceived risk to their infants. Practical considerations, such as the need to conduct home visits and maintain minimal interference with breastfeeding routines, create further obstacles to generating high-quality data on antiviral use during lactation [8]. The scarcity of predictive preclinical models and the limited utility of observational research and post-marketing surveillance methods frequently fail to provide comprehensive safety data [9].

As a result, lactating women have been systematically excluded from drug development pipelines and regulatory frameworks, leading to a pervasive lack of specific drug labeling information for this population [10]. While policy reforms, such as the U.S. Food and Drug Administration’s Pregnancy and Lactation Labeling Rule, have attempted to address these gaps, significant shortcomings remain [11]. Promising avenues to overcome these limitations include the development of pharmacokinetic modeling to estimate drug concentrations in breast milk and the strategic integration of lactation studies into early drug development phases [12,13].

Against this backdrop, the present systematic review aims to critically appraise the existing literature on the safety and efficacy of antiviral therapies used to manage Herpesviridae infections, including HSV, VZV, CMV, and EBV in lactating mothers. By collating and evaluating current evidence, this review seeks to illuminate best practices, identify research gaps, and ultimately guide future studies to ensure safer and more effective antiviral treatment options for both mothers and their infants.

## 2. Materials and Methods

This systematic review aims to evaluate the safety and efficacy of antiviral agents for lactating women with infections caused by the Herpesviridae family viruses, specifically Cytomegalovirus (CMV), varicella-zoster virus (VZV), Epstein–Barr virus (EBV), and herpes simplex virus (HSV) 1 and 2.

### 2.1. Research Question

The research question was formulated using the PICO framework as follows:

- Population: Lactating mothers with infections caused by Herpesviridae viruses (CMV, VZV, EBV, HSV).
- Intervention: Administration of antiviral agents during breastfeeding.
- Comparator: Lactating mothers with Herpesviridae infections not receiving antiviral therapy.
- Outcomes: Safety and efficacy of antiviral agents during lactation, potential prevention of viral transmission to the infant, changes in viral load in breast milk, and presence or alterations of protective factors in breast milk. In the initial phase, all identified outcomes will be documented. In the subsequent phase, the focus will primarily shift to evaluating the safety and efficacy of antiviral agents.

### 2.2. Search Strategy

A comprehensive literature search was conducted across three major databases: PubMed, Cochrane Library, and Scopus. Search queries were structured to retrieve studies related to breastfeeding, Herpesviridae infections, and antiviral agents. For PubMed, the search strategy incorporated a combination of Medical Subject Headings (MeSH) terms and free-text keywords, using Boolean operators and appropriate truncations to optimize the results. Similar search queries were adapted for the Cochrane Library and Scopus to account for database-specific indexing and syntax. The search strategy, which served as the basis for constructing tailored algorithms/queries for each database, is detailed in Supplementary Table S1 (Supplementary Materials). Additionally, all citations and references of the included articles were manually screened to identify relevant studies. Further, specialized databases such as LactMed were also scanned to ensure comprehensive coverage of the literature.

### 2.3. Inclusion and Exclusion Criteria

The following criteria were applied for study selection:

Inclusion criteria:

1. Studies involving lactating women with CMV, VZV, EBV, and HSV infections receiving any antiviral therapy.
2. Study types: Systematic reviews, randomized controlled trials (RCTs), clinical trials, observational studies, and case reports.
3. Outcomes of interest: Safety and/or efficacy of antiviral therapy, evaluation of Herpesviridae viral load in breast milk, presence of protective factors in breast milk, and related parameters.

Exclusion criteria:

1. Studies not involving or not reporting on lactating women.
2. Studies focused on viruses outside the Herpesviridae family (exceptions include studies addressing concurrent HIV and Herpesviridae infections).
3. Studies published in languages other than English.
4. Non-human studies.

### 2.4. Study Selection Process

**Figure 1:**
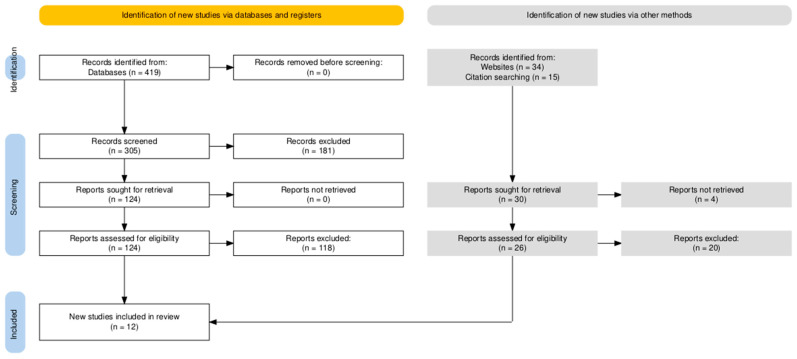
PRISMA Diagram.

The study selection process followed the PRISMA guidelines, with all identified records screened independently by two reviewers for eligibility based on the predefined criteria. Discrepancies were resolved by consensus or through consultation with a third reviewer. The PRISMA flow diagram (Figure 1) documents the screening and selection process, including reasons for exclusions.

### 2.5. Data Extraction

Data extraction was performed using a standardized form to systematically collect study characteristics (author, year, design, country), population details (sample size, age, lactation status, infection type), intervention specifics (antiviral type, dosage, duration), and outcomes (safety, efficacy, viral transmission, breast milk viral load, adverse events).

### 2.6. Quality Assessment

The quality of included studies was assessed using the Joanna Briggs Institute (JBI) quality assessment tools for case reports, cohort studies, and randomized controlled trials. Each included study was individually evaluated, and separate questionnaires were completed for each study category. Assessment results were recorded in structured tables, stratified by study type (RCTs, cohort studies, and case reports) (Supplementary Tables S2–S4).

### 2.7. Data Synthesis

A qualitative synthesis of the data was conducted to summarize findings across studies. The extracted data were organized into tables stratified by the study types identified, with one table for randomized controlled trials (RCTs) and another for case reports and case series.

## 3. Results

A total of twelve reports were included in this systematic review, comprising three RCTs, four observational studies, and four case reports. All clinical studies involve HIV-positive populations. Three of these studies provide evidence on the safety, efficacy, and pharmacokinetics of Valacyclovir in this patient population, while the rest offer insights into the impact of antiretroviral therapy (ART) per se on Herpetoviridae-HIV coinfected individuals. All included case reports focused on the use of acyclovir in lactating mothers, particularly for infections caused by VZV and HSV. The findings are summarized below, categorized by study type.

### 3.1. Clinical Studies

In total, eight clinical studies were identified to evaluate antiviral therapies’ efficacy and safety against Herpesviridae in lactating mothers (Table 1). Seven of these studies focused on CMV/HIV co-infected mothers, assessing virological outcomes such as CMV shedding in breast milk (quantified via PCR) and infant CMV acquisition (measured through IgG avidity assays or PCR). The eighth study, involving HSV/HIV co-infected mothers, was included for its evaluation of safety outcomes related to valacyclovir administration during lactation, including maternal and infant adverse events. No eligible clinical trials/studies investigating antiviral therapy for EBV or HSV in lactating women were identified. The complete search strategy, including terms used for these viruses, is provided in the Supplementary Materials.

Among the CMV/HIV studies, three investigated the impact of valacyclovir on CMV dynamics, used in conjunction with concurrent highly active antiretroviral therapy (HAART). These studies primarily assessed reductions in CMV shedding in breast milk and infant CMV acquisition. The remaining four CMV/HIV studies evaluated the effects of antiretroviral therapy (ART) alone, directly quantifying CMV viral load in breast milk or inferring transmission through infant seroconversion. Notably, these studies highlighted that while ART improved maternal immune reconstitution, it did not significantly reduce CMV levels in breast milk or prevent infant CMV acquisition.

In a randomized controlled trial by Roxby et al., the use of valacyclovir (500 mg twice daily) during late pregnancy and early lactation resulted in a modest reduction in cervical CMV shedding (0.4 log10 reduction, *p* = 0.05) [14]. However, no significant reduction in breast milk CMV loads was observed (valacyclovir: 5.4 log10 vs. placebo: 5.7 log10, *p* = 0.2). The drug was considered safe for lactating mothers and their infants, with minimal acyclovir exposure through breast milk and no major adverse effects reported. Meyer et al. assessed the effects of maternal HAART on HIV-1 and CMV transmission in a prospective cohort study [15]. The study found lower rates of HIV-1 transmission in the HAART group compared to untreated mothers (0% vs. 19%, *p* = 0.06). However, CMV loads in breast milk were not significantly reduced by HAART (*p* = 0.83). Importantly, higher CMV loads were associated with poorer infant growth outcomes, including lower length-for-age and weight-for-age Z-scores (*p* < 0.05). Slyker et al. further examined HAART in a randomized controlled trial, demonstrating that while it reduced infant CMV infection rates at 12 months compared to a Zidovudine-single dose Nevirapine regiment (75% vs. 94%, *p* = 0.04), it did not suppress CMV levels in breast milk, which remained detectable in nearly all samples (*p* = 0.01) [16]. Giuliano et al. observed that long-term ART (Option B-plus) did not reduce CMV DNA levels in breast milk (median 4.4 log₁₀ IU/mL at Months 1 and 12), nor did it prevent high infant CMV seroconversion rates (92.8% CMV IgG+ at 12 months) [17]. In their 2023 study, Giuliano et al. similarly found that maternal ART did not significantly reduce infant CMV acquisition, with 33.3% of HIV-exposed and 38.5% of HIV-unexposed infants testing CMV-positive at 6 months (*p* = 0.488) [18]. Although longer maternal ART duration (28 vs. 3 months) trended toward lower CMV positivity, this difference was not statistically significant (*p* = 0.187). Kourtis et al. also found no significant reduction in CMV transmission among HIV-exposed but uninfected infants, despite maternal ART or infant nevirapine, with 88.9% of infants acquiring CMV by 24 weeks postpartum [19]. Similarly, Pirillo et al. found that maternal ART (stavudine/zidovudine-based) during breastfeeding failed to prevent infant CMV acquisition, with 59.3% of infants CMV DNA-positive by 6 months and 96.4% seroconverted by 24 months, despite declining but persistent high CMV levels in breast milk (5.7 to 5.1 log10 IU/mL) [20].

Drake et al. conducted a randomized, double-blind, placebo-controlled trial in Kenya involving 148 HIV-1/HSV-2 co-infected pregnant women and their infants, assessing the effects of valacyclovir (500 mg orally, twice daily) from 34 weeks of gestation to 12 months postpartum [21]. Valacyclovir significantly reduced maternal plasma HIV-1 RNA levels (mean reduction of 0.56 log10, *p* < 0.001) and breast milk HIV-1 RNA loads compared to placebo. Acyclovir was detected in 80% of breast milk samples, with a median concentration of 2.62 µg/mL, resulting in an estimated infant exposure of ~0.39 mg/kg/day (~1% of the therapeutic pediatric dose), which is deemed safe. Although HSV shedding in breast milk was not assessed, no significant adverse effects were reported in mothers or infants. Infants in the valacyclovir group had lower rates of eczema (IRR 0.29, *p* = 0.02) and oral thrush (IRR 0.67, *p* = 0.05).

### 3.2. Case Reports

Four case reports were included that specifically addressed the use of acyclovir (as the sole antiviral agent assessed) in lactating mothers with Herpesviridae infections, providing evidence of its safety and clinical efficacy through detailed clinical examples—with cases primarily involving VZV and HSV infections (Table 2).

Meyer et al. investigated the pharmacokinetics of 200 mg oral acyclovir in a lactating mother with herpes zoster, reporting a breast milk-to-serum concentration ratio of 3.24 and an estimated infant exposure of 1 mg/day [22]. This was considered a low theoretical risk, supporting the safety of breastfeeding during maternal therapy in the absence of infant renal impairment. Taddio et al. similarly found that infant exposure to acyclovir was minimal (~1% of the maternal dose) during a regimen of 800 mg oral acyclovir administered five times daily for herpes zoster, with no observed adverse effects [23].

In cases of HSV infections, acyclovir therapy was shown to be both safe and effective. Agarwal et al. reported that a lactating mother was treated with systemic (400 mg orally, five times daily) and topical acyclovir for HSV-1 keratitis [24]. The infant remained unaffected during breastfeeding, and the mother experienced significant vision improvement after three months. In another case, Bork et al. described a mother with eczema herpeticum (likely HSV-1), a severe disseminated HSV infection, successfully treated with intravenous acyclovir (300 mg every 8 h) [25]. Breastfeeding, initially paused, was safely resumed 5–6 days post-treatment. Although acyclovir levels were higher in breast milk (7.3 µg/mL) than in serum (6.5 µg/mL), estimated infant exposure remained below toxic thresholds, and no herpes virus was detected in breast milk, further affirming breastfeeding safety after maternal treatment.

### 3.3. Quality Assessment Results

The included RCTs demonstrated moderate-to-high methodological quality. All trials used true randomization with balanced baseline characteristics and complete follow-up. Outcomes were reliably measured, and statistical methods were appropriate. However, some trials lacked allocation concealment details or had incomplete blinding (participants/personnel), introducing potential performance/detection bias. Despite these limitations, methodological rigor (consistent outcome measurement, intent-to-treat analysis) supported credible findings.

The included cohort studies demonstrated moderate methodological quality. All studies measured exposures and outcomes reliably, with sufficient follow-up duration. However, handling of confounders varied, as only one study identified and adjusted for confounders, while others either partially addressed or omitted them. Attrition bias was noted in four studies due to incomplete follow-up reporting. Despite these limitations, statistical methods were appropriate, supporting the validity of findings for synthesis.

The quality assessment of the included case reports revealed consistent strengths and some notable limitations. All case reports clearly describe patient demographics, clinical history (including timelines), presenting conditions, and diagnostic tests or methods used. Furthermore, each report provided meaningful takeaway lessons, contributing valuable insights into the systematic review. One case report was excluded as it did not provide any information on the treatment details and outcomes of the mother [26]. Additionally, two reports did not adequately describe the post-intervention clinical condition, and adverse events were inconsistently reported, with only one case report addressing them.

Quality assessment tables can be found in the Supplementary Materials (Supplementary Tables S2–S4).

## 4. Discussion

We performed a systematic review on the efficacy and safety of antivirals in lactating women with Herpesviridae infections. It appears that antiviral treatments like valacyclovir and acyclovir are considered safe for use in breastfeeding mothers, as they result in minimal infant exposure and carry a very low risk of adverse effects. Nonetheless, they offer modest virologic benefits, while there is limited availability of comprehensive clinical trials across diverse populations.

Antiviral medications during lactation require careful consideration due to potential risks to nursing infants. While some antivirals are considered safe, others are contraindicated. Generally, clinicians should consider individual patient factors when prescribing antivirals to lactating women and monitor for potential adverse effects [27], taking into consideration factors such as milk concentrations, milk-to-plasma ratios, and available safety data to minimize risks to nursing infants. While these studies collectively suggest that valacyclovir and antiretroviral therapy can be administered with acceptable safety profiles to both mothers and infants, the degree to which antiviral interventions effectively reduce Herpesviridae viral loads in breast milk remains less clear. The impact observed in certain virologic outcomes can be deemed modest at best. However, the subtle virologic changes observed in some studies come with the possibility that standard antiviral doses—chosen for their established safety margins—may be insufficient to alter breast milk transmission dynamics of these viruses substantially. Notably, prior research on CMV vertical transmission prevention has investigated significantly higher doses of valacyclovir (e.g., 4000 mg BID) [28], leaving uncertainty about whether lower doses, such as the 500 mg BID regimen commonly used during lactation trials, could achieve similar effects.

Apart from the pharmacokinetic parameters that need to be considered, a pre-ART breastfeeding study [29] provides important epidemiological benchmarks and insights that could help inform treatment and/or prophylaxis strategies during lactation. This study demonstrated that breastfeeding nearly doubled infant CMV acquisition risk (HR 1.6, 95% CI 1.2–2.2) and significantly accelerated infection timing (median 4.3 vs. 9.9 months) compared to formula feeding. Importantly, it quantified three distinct transmission routes: peripartum (33%), breast milk-associated (40%), and other postnatal exposures (27%), with breast milk transmission occurring predominantly during the first 4 months postpartum. These findings establish clear targets for antiviral interventions—both in terms of the preventable transmission fraction (40%) and the critical intervention window (0–4 months).

A key limitation of these data is that they are predominantly derived from HIV-positive cohorts—populations most frequently exposed to antivirals—making the findings especially pertinent to HIV-infected lactating women. However, this focus may not fully capture the pharmacokinetics, safety, and efficacy of these antiviral therapies in HIV-negative women with Herpesviridae infections. While some studies suggest that pharmacokinetic parameters may differ between HIV-positive and HIV-negative individuals due to variations in immune status, drug metabolism, and viral dynamics, most of the available evidence on such differences comes from research on protease inhibitors [30]. Thus, it remains unclear whether similar differences exist for antivirals such as acyclovir and valacyclovir. Moreover, the modest virologic benefits observed in HIV-positive cohorts may not directly translate to HIV-negative populations, where immune responses to Herpesviridae infections might be more robust.

The findings from the identified case reports collectively suggest the safety of acyclovir in treating Herpesviridae-caused infections in lactating mothers while allowing breastfeeding to continue, except when direct contact with active lesions posed a risk of transmission (e.g., HSV lesions on the breast). Across these cases, a consistent theme emerged: acyclovir is excreted into breast milk in low concentrations, leading to minimal exposure for the infant, which was deemed clinically insignificant in all instances. Moreover, no adverse effects were reported in any of the breastfed infants, implying the safety of continuing breastfeeding during antiviral therapy. Temporary interruptions in breastfeeding, as seen in Bork et al. [25], were primarily due to risks associated with direct contact with active lesions rather than systemic drug safety concerns. The majority of the included case reports indicate that treatment durations of 5–7 days are adequate for managing acute infections (e.g., herpes zoster and eczema herpeticum). Only one case report involved long-term treatment lasting several months for herpes simplex keratitis, during which the baby, after 3 months of breastfeeding, showed no signs of systemic herpetic infection or treatment-related adverse effects. Despite these findings, the lack of consistent assessments of viral presence in breast milk and limited long-term infant follow-up in the included cases leaves some areas open for future research.

On the other hand, the minimal adverse effects and low-level infant exposure to antiviral agents observed in the included studies provide a measure of reassurance. These findings support the notion that, under specific circumstances and with appropriate safeguards, it is possible to balance the necessity of antiviral therapy in lactating mothers against the imperative to minimize risks to the nursing infant. Nonetheless, the scarcity of robust clinical trials in this area remains a critical gap. Extending research efforts beyond the HIV co-infected population, employing more refined virologic and clinical endpoints, and exploring alternative antivirals or combination therapies will be essential to guide evidence-based decision-making for lactating women with Herpesviridae infections. There is also a need for further research to determine optimal dosing strategies and to clarify the clinical significance of potentially modest viral load reductions.

In this context, the prospective pharmacokinetic study by Sheffield et al. provides valuable insights, despite being excluded from the review due to the absence of Herpesviridae coinfection in the studied population [31]. In this study, five breastfeeding women received valacyclovir (500 mg orally, twice daily) for seven days, and matched maternal serum and breast milk samples were analyzed. Acyclovir, the active metabolite of valacyclovir, was found at consistently higher concentrations in breast milk than in serum, with a peak concentration of 4.2 µg/mL at 4 h in breast milk compared to 2.7 µg/mL at 1 h in serum. The steady-state milk concentration was 2.24 µg/mL, with a milk-to-serum ratio of 1.85. Estimated infant drug exposure was calculated at 0.61 mg/kg/day, approximately 2% of the therapeutic neonatal dose (30 mg/kg/day), and was deemed negligible and safe. Due to the low oral bioavailability of acyclovir in infants, systemic exposure was further reduced to less than 0.5% of the intravenous therapeutic dose. No HSV was detected in breast milk, and no adverse effects were observed in mothers or infants. The case reports also provide insights into the pharmacokinetics of acyclovir during lactation. For example, Taddio et al. observed that oral acyclovir concentrations in breast milk peaked at 30 h post-treatment and remained detectable for 4 days, while serum concentrations declined more rapidly [23]. Moreover, Meyer et al. calculated a half-life of 2.8 h for acyclovir in breast milk, although at a low dosage (200 mg per day PO) [22].

This comes in line with results from other viral infections and respective antivirals. In influenza patients, Fodor et al. estimated oseltamivir infant exposure through breast milk to be less than 0.1% of the therapeutic infant dose [32], with a relative infant dose (RID) of approximately 0.5% of the maternal weight-adjusted dose [33]. These low concentrations are not expected to cause clinically significant effects in breastfed infants [33,34]; however, as for other antivirals, more research is needed to confirm the safety of oseltamivir during breastfeeding [34].

Similarly, in the context of SARS-CoV-2, remdesivir appears to have low milk concentrations and poor oral absorption, suggesting minimal risk to breastfed infants [35]. However, careful monitoring is still advised [36]. Current guidelines recommend nirmatrelvir/ritonavir as an outpatient treatment option for lactating individuals with mild COVID-19 [37]. While clinical data on nirmatrelvir/ritonavir use during pregnancy and lactation are limited, similar to others, animal studies have shown no significant teratogenicity or adverse effects on fertility and early embryonic development [38]. A cross-sectional study of pregnant and lactating individuals who took nirmatrelvir/ritonavir reported no significant adverse outcomes, while the safety profile of ritonavir in pregnant HIV patients provides additional reassurance [39]. In the HIV setting, the breast milk to maternal plasma ratios for nucleoside reverse transcriptase inhibitors (NRTIs) range from 0.89 to 1.21, non-nucleoside reverse transcriptase inhibitors (NNRTIs) from 0.71 to 0.94, and protease inhibitors (PIs) from 0.17 to 0.21 [40]. Infants may ingest up to 12.5% of the recommended pediatric dose of nevirapine through breast milk [40]. Nonetheless, nevirapine concentrations in infant serum can reach levels similar to therapeutic doses, potentially exposing infants to both beneficial and adverse effects [41].

Studies on tenofovir during lactation have shown that it passes into breast milk at low levels. In chronic hepatitis B patients, tenofovir concentrations in breast milk were approximately 7% of maternal plasma levels, with undetectable levels in infant plasma [42], while another study on HBV-infected mothers found low tenofovir concentrations in breast milk, ranging from 1.4 to 11.7 ng/Ml [43]. Animal studies in rhesus macaques corroborate these findings, with milk concentrations about 3% of serum levels [44]. Importantly, infant growth parameters were normal in breastfed infants of mothers receiving tenofovir [43]. These studies suggest that tenofovir use during breastfeeding results in minimal infant exposure and is likely safe for both chronic hepatitis B treatment and HIV prevention. The same does not apply for direct-acting antivirals (DAAs) that have currently revolutionized hepatitis C virus (HCV) treatment, offering high efficacy and tolerability [45]. Their use during pregnancy and lactation remains controversial and is currently not recommended due to insufficient safety information [46,47,48].

This review is subject to several limitations. First, the majority of included clinical trials were conducted in HIV-positive populations, restricting the generalizability of findings to lactating women without HIV co-infection. The available clinical trials were also marked by a remarkable absence of studies evaluating antiviral therapy for EBV in lactating women. This may be explained by the fact that EBV infections in immunocompetent individuals are typically self-limited and rarely require pharmacological treatment [49], limiting the clinical need for such research. Nonetheless, lactation has been identified as a potential transmission route for EBV and warrants further investigation [50]. Additionally, most studies reported only short-term outcomes, with limited data on long-term infant safety or developmental impacts. The exclusion of trials lacking direct lactation-related outcomes may have overlooked valuable secondary insights. Finally, the heterogeneity in study designs and endpoints, including inconsistent assessments of viral presence in breast milk, complicates direct comparisons between studies and limits the synthesis of findings.

On another note, this review confirms that research on antiviral use during lactation remains limited, reflecting the historical underrepresentation of lactating women in clinical drug development. Ethical and logistical challenges contribute significantly to this gap. Ethical concerns primarily stem from the potential unintended drug exposure of infants via breast milk, necessitating careful risk–benefit considerations [51]. Additionally, obtaining informed consent in this context is complex, as it must account for both maternal autonomy and infant safety [52]. Logistical barriers, including variability in breastfeeding practices, difficulties in collecting breast milk and infant blood samples, and differences in maternal drug metabolism, further complicate study design and data collection [53]. These challenges have led to a scarcity of well-powered clinical trials, ultimately limiting the available pharmacokinetic and safety data for many medications, including antivirals. Recognizing these obstacles, recent initiatives, such as the Task Force for Research Specific to Pregnant Women and Lactating Women, advocate for a shift in research priorities to improve the inclusion of this population in clinical studies [54]. Future research should aim to address these limitations through innovative study designs that ensure both ethical integrity and scientific accuracy [55].

## 5. Conclusions

To conclude, the safety and efficacy of antivirals during lactation remain underexplored, particularly for Herpesviridae infections in the absence of HIV co-infection. Current evidence suggests that antiviral therapies such as valacyclovir and acyclovir can be used safely in lactating mothers, with negligible infant exposure and minimal risk of adverse effects. However, the modest virologic benefits observed, coupled with the scarcity of robust clinical trials in diverse populations, highlight the need for further research on the subject. Future studies should incorporate pharmacokinetic modeling to refine dosing strategies and evaluate drug distribution in breast milk and infant plasma. Additionally, well-designed observational cohort studies could provide critical insights into long-term outcomes and the real-world impact of antiviral use during breastfeeding. Pharmacokinetic studies are needed to refine dosing strategies and achieve optimal antiviral efficacy in lactating women. Subsequently, well-designed observational studies with clearly defined clinical endpoints—and, where feasible, long-term follow-up—are necessary to provide accurate data on the real-world clinical efficacy of these treatments.

## Figures and Tables

**Table 1:** Data Extraction Table for Clinical Trials/Studies.

Author(s) Year Country	Study Design	Sample Size	Participant Characteristics (Age, Lactation Status)	Virus Type	Antiviral Used	Dosage	Duration of Treatment	Comparator Group	Outcome(s) (Safety, Efficacy)	Virus Presence in Breast Milk	Adverse Effects	Primary Findings, Conclusions
Roxby et al. 2014 [14] Kenya	Randomized, double blind, placebo controlled trial	− 147 women; − 141 infants analyzed for CMV	Pregnant HIV-1/HSV-2 coinfected women; median age 25 years (IQR 22–29); CD4 count >250 cells/μL; WHO stage 1 or 2 HIV; exclusively breastfeeding for 6 months postpartum	CMV HIV-1	Valacyclovir	500 mg orally, twice daily	From 34 weeks gestation to 12 months postpartum	Placebo group; both groups received standard PMTCT prophylaxis (maternal zidovudine from 28 weeks, maternal and infant nevirapine at delivery, and 6 weeks of infant zidovudine).	− Similar between arms (66% in both placebo and valacyclovir arms; *p* = 1.0). − Cervical CMV shedding modestly reduced in valacyclovir arm (0.4 log_10_ copies/mL reduction; *p* = 0.05). No significant difference in breast milk CMV levels (placebo: 5.7 log_10_ vs. valacyclovir: 5.4 log_10_, *p* = 0.2). − No significant differences in adverse events or hospitalizations; infant growth similar (weight-for-age Zscores: −0.35 placebo vs. −0.31 valacyclovir; *p* = 0.85).	− CMV detected in 92% of breast milk samples; − Valacyclovir did not reduce CMV levels in breast milk.	No major adverse effects in mothers or infants.	− Maternal valacyclovir modestly reduced cervical CMV shedding during late pregnancy but did not impact breast milk − CMV levels or timing of infant CMV acquisition. − Infant exposure to acyclovir through breast milk was minimal and deemed safe. − Higher doses of valacyclovir or alternative antivirals may be required to significantly affect CMV transmission.
Meyer et al. 2014 [15] Malawi	Prospective cohort study	− 69 HIV infected lactating women; − 55 HIV exposed, uninfected infants (after excluding infected and deceased infants)	− HIV-1-infected, CMV-seropositive lactating women enrolled at delivery; recruited via rapid antibody testing, confirmed by CD4+ T-cell count (median 315–382 cells/μL) and plasma HIV-1 RNA viral load in third trimester; CMV seropositivity by third trimester IgG ELISA. − HAART group mean age 33 vs. untreated group mean age 26 (*p* = 0.0002). Follow up to six months postpartum, breast milk samples at 4−6 weeks for viral load analysis; − HIV−exposed, uninfected, breastfed infants, ages birth to six months; HIV-1 testing via PCR at birth, 4–6 weeks, every three months; congenital CMV status via cord blood PCR and IgM.	CMV HIV-1	HAART (Trioimmune: stavudine, lamivudine, nevirapine)	Not specified for each drug	Varied start time − 16 women on therapy prior to enrollment, − 14 initiating HAART during pregnancy and 2 on therapy prior to pregnancy	− Un-treated HIV− infected women, received single-dose nevirapine at delivery; − Infants with single-dose nevirapine at birth	− Increased low birth weight in HAART group (25%) vs. untreated group (2%), *p* = 0.009 − HAART associated with lower in utero or postpartum HIV-1 transmission (0% HAART group vs. 19% untreated, *p* = 0.06). − Higher CMV load in breast milk associated with reduced infant length-for-age (−0.53, *p* < 0.05) and weightfor-age Z-score (−0.40, *p* < 0.05) at 6 months.	− CMV DNA detected in breast milk of both HAART− treated and untreated women, with no significant difference in CMV load (*p* = 0.83). − Minimal effect of HAART on CMV load and no association with subclinical mastitis. − Higher CMV loads in HIV transmitting mothers vs. nontransmitting, *p* = 0.003. − Weak association between CMV and HIV-1 loads in milk (0.39 log_10_ CMV increase per 1 log_10_ HIV increase, *p* < 0.05), indicating co-shedding but no direct correlation.	− Low birth weight more common in HAART group (25%) vs. untreated group (2%), *p* = 0.009; − No significant association between preterm birth and CMV infection detected (*p* = 0.82).	− Maternal HAART did not significantly reduce CMV load in breast milk but was associated with lower HIV transmission; − Higher CMV load in breast milk was linked to poorer infant growth outcomes.
Slyker et al. 2017 [16] Kenya	Randomized controlled trial	51 women (26 HAART, 25 ZDV/sdNVP); and their infants	HIV-positive, antiretroviral-naïve pregnant women; CD4 200–500 cells/mm³; Median age 26 years (ZDV/sdNVP group) and 24 years (HAART group).; breastfeeding through 6 months postpartum	CMV HIV-1	HAART: zidovudine + lamivudine + nevirapine; ZDV/sdNVP: zidovudine + single-dose nevirapine	HAART: 300 mg zidovudine + 150 mg lamivudine + 200 mg nevirapine (twice daily); ZDV/sdNVP: 300 mg zidovudine (twice daily antenatally; every 3 h during labor) + maternal/infant nevirapine at delivery	HAART: 34 weeks gestation to 6 months postpartum; ZDV/sdNVP: 34 weeks gestation until delivery	ZDV/sdNVP (short-course antenatal zidovudine + single-dose nevirapine for PMTCT)	− Efficacy: HAART reduced infant CMV infection probability at 12 months (75% vs. 94% in ZDV/sdNVP; *p* = 0.04). − Safety: No major adverse effects reported; adherence was high in both arms. Three infants acquired HIV (2 HAART, 1 ZDV/sdNVP).	− CMV detected in 100% of breast milk samples; 72% positive in first week, 98% by second week. − HAART did not reduce CMV levels (slower decline vs. ZDV/sdNVP; *p* = 0.01).	None reported	HAART initiated in third trimester reduced vertical CMV transmission by 19% compared to ZDV/sdNVP, but did not reduce breast milk CMV levels. Reduction in transmission likely due to systemic immune effects rather than breast milk CMV suppression.
Guiliano et al. 2017 [17] Malawi	Observational cohort	30 mothers; 14 infants tested	HIV-positive pregnant women; median age 27.5 years (IQR 23–31.3); median ART duration: 17 weeks during pregnancy + 1 year postpartum	CMV, HIV-1	Tenofovir + Lamivudine + Efavirenz	Not specified	ART started during pregnancy (median 121.5 days) and continued for 1 year postpartum	None (observational)	− Efficacy: 92.8% (13/14) infants CMV IgG+ at 12 months; breast milk CMV DNA stable at 4.4 log_10_ IU/mL (Month 1 and 12). − Safety: Not explicitly reported	CMV detected in 100% of breast milk at Month 1 and 93.3% at Month 12 (2/30 undetectable). Median CMV load: 4.4 log_10_ IU/mL at both timepoints.	None reported	− Long-term ART (Option B-plus) did not reduce breast milk CMV DNA levels or infant CMV acquisition (92.8% infected by 12 months). − Immune reconstitution (CD4 up, from 469 to 637 cells/mm³) failed to suppress CMV shedding. ART duration had no correlation with CMV load.
Giuliano et al. 2023 [18] Malawi	Observational cohort	58 infants (45 HIV-exposed, 13 HIV-unexposed)	HIV-positive and HIV-negative mothers; median age 30 years (IQR 24–33); infants followed until 12 months postpartum; exclusive breastfeeding encouraged for 6 months	CMV HIV	TDF/3TC/EFV (91.1%) or TDF/3TC/DTG (8.9%) during pregnancy and breastfeeding	Not specified	− Median ART duration at enrollment: 6 months (IQR 3–88) − ART was initiated during pregnancy and continued through breastfeeding (exclusive breastfeeding encouraged for 6 months)	−HIV-exposed: 33.3% (15/45) CMV+. −HIV-unexposed: 38.5% (5/13) CMV+ (*p* = 0.488).	− No difference in CMV acquisition between HIV-exposed and HIV-unexposed infants. − Longer maternal ART duration (28 vs. 3 months) trended toward lower CMV positivity (*p* = 0.187).	Not measured (breast milk CMV not tested)	No impact on infant growth or infectious events.	− Maternal ART did not significantly reduce infant CMV acquisition. − Vaginal delivery associated with CMV positivity (*p* = 0.015). − Mothers of CMV-negative infants had a longer median ART duration (28 months) compared to mothers of CMV-positive infants (3 months), though this difference was not statistically significant (*p* = 0.187).
Kourtis et al., 2015 [19] Malawi	Observational sub-study (RCT)	28 infants (birth), 127 (24 weeks), 107 (48 weeks)	HIV-exposed, uninfected infants (birth weight ≥2000 g); mothers randomized to: − Maternal triple-drug ART − Infant nevirapine − Control (1-week postpartum ART)	CMV	Maternal ART (Zidovudine + Lamivudine + Nevirapine) or infant Nevirapine vs. control (short-course ART)	Not specified	28 weeks of breastfeeding (intervention arms)	− Birth: 8/28 (28.6%) CMV DNA+ (congenital). − 24 weeks: 88.9% (113/127) CMV-infected (serology + PCR). − 48 weeks: 81.3% (87/107) CMV IgG+ (infant seroconversion).	− Maternal ART did not reduce infant CMV infection. − No difference in CMV acquisition between maternal ART, infant nevirapine, or control arms.	Not measured (breast milk CMV not tested)	None reported	− High CMV acquisition (89% by 24 weeks) despite maternal/infant ART during breastfeeding. − Maternal ART failed to reduce CMV transmission. − CMV IgG avidity increased with age (19% high avidity at 24 weeks vs. 71% at 48 weeks).
Pirillo MF et al. 2017 [20] Malawi	Observational cohort	89 mother-child pairs	HIV-positive mothers; median age 26 years (IQR 23–30); baseline CD4: 333 cells/mm³; all CMV IgG+ at baseline	CMV, HIV-1	Maternal ART: Stavudine or Zidovudine + Lamivudine + Nevirapine	Not specified	ART started at 25 weeks gestation; continued for 6 months postpartum (or indefinitely if CD4 < 350 cells/mm³)	− 6 months: 59.3% (53/89) CMV DNA+ in infant plasma. − 12 months: 47.6% (40/84) CMV DNA+. − 24 months: 96.4% (80/83) CMV IgG+.	− CMV DNA levels in breast milk: 5.7 log_10_/mL IU/mL at Month 1, declining to 5.1 log_10_/mL IU/mL at Month 6 (*p* = 0.001). − HIV-infected infants had higher CMV DNA levels at 12 months (3.3 vs. 2.3 log_10_/mL, *p* = 0.001). − No impact on infant growth.	CMV DNA detected in breast milk: 5.7 log 10/mL IU/mL at Month 1, 5.1 log 10/mL IU/mL at Month 6.	None reported	− Maternal ART did not prevent infant CMV acquisition. − Breast milk CMV levels declined over time but remained high. − HIV-infected infants had higher CMV DNA levels. − Low maternal socioeconomic status correlated with infant CMV infection (*p* = 0.036).
Drake et al. 2012 [21] Kenya	Randomized, double blind, placebo controlled trial	148 HIV-1/HSV-2 co-infected pregnant women; 146 mother infant pairs followed postpartum	Pregnant women (median age 25 years, IQR 22–29) with CD4 count >250 cells/mm³; breastfeeding for a median of 5–6 months postpartum	HIV-1 HSV-2	Valacyclovir	500 mg orally, twice daily	From 34 weeks gestation to 12 months postpartum	Placebo group receiving standard PMTCT ARVs (Zidovudine at 28 weeks, single-dose Nevirapine at delivery, and postnatal ARVs for infants)	− No significant differences in infant creatinine (median 0.50 mg/dL) or ALT (median 26.5 U/L), both within normal ranges. Infant growth (weight-for-age Zscores: −0.31 vs. −0.35, *p* = 0.85) and hospitalization rates (7 in placebo vs. 2 in valacyclovir, HR 0.27, 95% CI 0.06–1.32, *p* = 0.11) were similar between groups. − Reduced maternal plasma HIV-1 RNA levels and breast milk HIV-1 RNA load compared to placebo. − Median acyclovir concentration in breast milk: 2.62 µg/mL; estimated infant exposure ~0.39 mg/kg/day (1% of therapeutic pediatric dose), deemed safe.	Acyclovir detected in 80% of breast milk samples; HSV not assessed in breast milk.	− No major maternal or infant toxicities reported − Lower incidence of eczema and oral thrush in infants from the valacyclovir group.	− Valacyclovir significantly reduced maternal plasma HIV-1 RNA levels (mean reduction of 0.56 log10, *p* < 0.001) and breast milk HIV− 1 RNA load compared to placebo. − Infant eczema and oral thrush were lower in the valacyclovir group (IRR for eczema: 0.29, *p* = 0.02; IRR for oral thrush: 0.67, *p* = 0.05). − Overall, valacyclovir exposure was safe for mothers and infants and effective in reducing maternal HIV-1 RNA levels during pregnancy and breastfeeding.

Abbreviations: CMV: Cytomegalovirus; HIV: Human Immunodeficiency Virus; HSV-2: Herpes Simplex Virus Type 2; IQR: Interquartile Range; CD4: Cluster of Differentiation 4; WHO: World Health Organization; PMTCT: Prevention of Mother-To-Child Transmission; RNA: Ribonucleic Acid; IgG: Immunoglobulin G; ELISA: Enzyme-Linked Immunosorbent Assay; HAART: Highly Active Antiretroviral Therapy; PCR: Polymerase Chain Reaction; IgM: Immunoglobulin M; DNA: Deoxyribonucleic Acid; ZDV/sdNVP: Zidovudine/single-dose Nevirapine; ART: Antiretroviral Therapy; TDF/3TC/EFV: Tenofovir disoproxil fumarate/Lamivudine/Efavirenz; TDF/3TC/DTG: Tenofovir disoproxil fumarate/Lamivudine/Dolutegravir; ARVs: Antiretrovirals; ALT: Alanine Aminotransferase; HR: Hazard Ratio; CI: Confidence Interval; IRR: Incidence Rate Ratio.

**Table 2:** Data Extraction Tables for Case Reports.

Author(s) Year Country	Case Details	Age of Participa nt(s)	Lactation Status	Virus Type	Antiviral Used	Dosage	Duration of Treatment	Outcome(s) (Safety, Efficacy)	Presence of Virus in Breast Milk	Key Findings/Conclusions
Meyer et al. 1988 [22] USA	Lactating mother, gravida 2 para 2, with a history of chickenpox at age 4 and treated with oral acyclovir for herpes zoster (dermatomes C6 to T3). Milk and serum specimens obtained during and after the final dose and acyclovir assayed with radioimmunoassay to study relative concentrations in serum and milk and half life of the drug during the elimination phase.	31 years old	one year postpartum	VZV	Acyclovir	200 mg PO/day	5 days	Estimated hypothetical dose consumed by the nursing baby crudely estimated from milk concentrations of 1.06 μg/mL multiplied by L/day milk production and consumption to a dose of 1 mg/day, which is of low theoretical risk given the normal renal function of the baby	Not Assessed	Estimated ratio of average concertation of acyclovir in breast milk and serum = 3.24 (breast milk = 1.06, serum = 0.33), half-life of acyclovir in breast milk was calculated as 2.8 h after the final dose, oral acyclovir in nursing mothers is of low theoretical risk provided normal renal function, no available acyclovir nursing guidelines at the time of publication
Taddio et al. 1994 [23] Canada	Lactating mother gravida 1, para 1 with herpes zoster virus infection contacting an antenatalperinatal counseling program to inquire about drug safety while lactating; milk samples analyzed for acyclovir concentration using radioimmunoassay	28 years old Infant of 7 months	Exclusively breastfeeding, continued during therapy	VZV	Acyclovir	800 mg PO, five times/day	7 days	Acyclovir concentrations in breast milk ranged from 18.5 µmol/L (4.16 µg/mL) to 25.8 µmol/L (5.81 µg/mL). Infant exposure estimated at 0.73 mg/kg/day (~1% of maternal dose); no adverse effects observed in the infant. No reported maternal adverse effects.	Not Assessed	Acyclovir excreted into breast milk in low concentrations; infant exposure through breastfeeding deemed clinically insignificant. Safe to continue breastfeeding during maternal acyclovir therapy.
Agarwal et al. 2019 [24] India	Lactating underweight, anemic woman with herpes simplex keratitis (HSK), confirmed by HSV PCR	22 years old	Lactating already for 2 months by symptom presentation	HSV-1	Acyclovir	400 mg PO 5 times per day + topical acyclovir 3% 5 times per day, initiated before PCR results due to clinical suspicion	Systemic acyclovir for at least 1 year	After 3 months of breastfeeding the baby showed no signs of systemic herpetic infection, maternal vision was improved	Not assessed	Lactation aggravated malnutrition and anemia may trigger reactivation of ocular herpes, HSK is not a contraindication to breastfeeding unless there are active lesions on the breast, no side effects of acyclovir on the baby
Bork et al. 1995 [25] Germany	Lactating woman with long-standing atopic dermatitis developing labial herpes simplex infection that spread to head and upper extremities (eczema herpeticum) within 5 days, fever (39.2 °C), malaise and fatigue.	29 years old	Breastfeeding interrupted, resumed postrecovery	HSV (most likely HSV-1)	Acyclovir	900 mg/day IV (300 mg every 8 h)	5 days	Resolution of maternal symptoms. Infant remained asymptomatic throughout, estimated acyclovir exposure through milk (1.3 mg/kg/day) would have been well below toxic levels.	No herpes virus detected in breast milk samples analyzed	Acyclovir concentrations higher in breast milk (7.3 μg/mL) than serum (6.5 μg/mL), detectable in milk longer (88 h or approximately 4 days) and reaching peak 30 h posttreatment while undetectable in serum after 40 h (approximately 2 days). Safe resumption of breastfeeding 5–6 days post-treatment; theoretical acyclovir exposure from breastfeeding considered safe for infants.

Abbreviations: VZV: Varicella-Zoster Virus; PO: Per Os; HSV: Herpes Simplex Virus; PCR: Polymerase Chain Reaction; HSK: Herpes Simplex Keratitis; IV: Intravenous.

## Data Availability

Data is contained within the article.

## Supplementary Materials

The following supporting information can be downloaded at: https://www.mdpi.com/article/10.3390/v17040538/s1, Supplementary Table S1: Search Strategy Details; Supplementary Table S2: Quality Assessment of Randomized Controlled Trials; Supplementary Table S3: Quality Assessment of Cohort Studies; Supplementary Table S4: Quality Assessment of Case Reports.
