# Supplementary material for: Efficacy and Safety of Antivirals in Lactating Women with Herpesviridae Infections: A Systematic Review
Source: Viruses. 2025 Apr 7;17(4):538. doi: 10.3390/v17040538 (PMC12031584; doi:10.3390/v17040538)
Supplement: Supplementary file 1 [file viruses-17-00538-s001.zip › viruses-3448894-supplementary.pdf]

Efficacy and Safety of Antivirals in Lactating Women with Herpesviridae Infections: A Systematic Review

Vasiliki Kallia [1,2,†], Georgios Schinas [3,4,*,†], Georgios Karagiannopoulos [2] and Karolina Akinosoglou [3,5]

1 Fourth of Internal Medicine, University General Hospital Attikon, Chaidari, 12462 Athens, Greece

2 National and Kapodistrian University of Athens, Vasilissis Sofias 98, 11528 Athens, Greece

3 School of Medicine, University of Patras, 26504 Rio, Greece

4 First Department of Internal Medicine, Laiko General Hospital, School of Medicine, National and Kapodistrian University of Athens, 11527 Athens, Greece

5 Department of Internal Medicine and Division of Infectious Diseases, University General Hospital of Patras, 26504 Rio, Greece

* Correspondence: georg.schinas@gmail.com

† These authors contributed equally to this work.

Supplementary Materials

**Supplementary Table S1. Search strategy for lactation and Herpesviridae infections treated with antiviral agents**
**#1: Lactation-related terms**<br>Lactation OR Breast Feeding OR Milk, Human OR Lactation* OR Milk Secretion* OR Human Milk* OR Breast Milk* OR Breastfeeding OR Breast Fed OR Breastfed OR Milk Sharing* OR Wet Nursing* OR Exclusive Breastfeeding* OR Prolonged Lactation* OR Milk*
**#2: CMV-related terms**<br>Cytomegalovirus Infections OR Cytomegalovirus OR Cytomegalovirus Infection* OR Cytomegalovirus Inclusion Disease* OR Cytomegalic Inclusion Disease* OR Perinatal Cytomegalovirus Infection* OR Perinatal CMV Infection* OR Congenital Cytomegalovirus Infection* OR Cytomegalovirus Inclusion* OR CMV Inclusion*
**#3: VZV-related terms**<br>Chickenpox OR Herpesvirus 3, Human OR Chickenpox Virus* OR Human Herpesvirus 3* OR HHV-3* OR Varicella-Zoster Virus* OR Varicella Zoster Virus* OR Varicella-Zoster Viruses OR VZ Virus* OR VZ Viruses OR Herpesvirus Varicellae* OR Herpes Zoster Virus* OR Herpes Zoster Viruses OR Chicken Pox* OR Varicella*
**#4: HSV-related terms**<br>Herpesvirus 1, Human OR Herpesvirus 2, Human OR HHV-1 OR Herpes Simplex Virus Type 1 OR Herpes Simplex Virus 1 OR HSV-1 OR Herpes Simplex Virus Type 2 OR Human Herpesvirus 2 OR HHV-2 OR HSV-2 OR Herpes Simplex Virus 2 OR Herpesvirus*
**#5: EBV-related terms**<br>Epstein-Barr Virus Infections OR Herpesvirus 4, Human OR Epstein-Barr Virus Infection*

OR Epstein Barr Virus Infection* OR EBV Infection* OR Human Herpesvirus 4 Infection* OR Human Herpes Virus 4 Infection*
**#6: Antiviral-related terms**<br>Antiviral Agents OR Antiviral Agent* OR Agents, Antiviral* OR Antivirals* OR Antiviral* OR Antiviral Drugs* OR Drugs, Antiviral* OR Antiviral Drug* OR Drug, Antiviral* OR Antiviral Therap* OR Antiviral Treatment* OR Viral Inhibitor* OR Viral Resistance* OR Broad-Spectrum Antivirals* OR Direct-Acting Antiviral* OR Host-Targeted Agents* OR Virus Replication Inhibitor* OR Viral Load Reduction* OR Viral Infection Treatment* OR RNA Polymerase Inhibitor* OR Protease Inhibitors* OR Entry Inhibitor* OR Fusion Inhibitor* OR Antiretroviral Therapy, Highly Active*
**Search Combinations:**<br>1. CMV: #1 AND #2 AND #6<br>2. VZV: #1 AND #3 AND #6<br>3. HSV: #1 AND #4 AND #6<br>4. EBV: #1 AND #5 AND #6

Supplementary Table S2. Quality Assessment of Randomized Controlled Trials

Study	Drake et al.	Roxby et al.	Slyker et al.
Was true randomization used for assignment of participants to treatment groups?	Yes	Yes	Yes
Was allocation to treatment groups concealed?	Yes	Yes	Unclear
Were treatment groups similar at the baseline?	Yes	Yes	Yes
Were participants blind to treatment assignment?	Yes	Unclear	No
Were those delivering treatment blind to treatment assignment?	Yes	Unclear	No
Were outcomes assessors blind to treatment assignment?	Yes	Unclear	Unclear
Were treatment groups treated identically other than the intervention of interest?	Yes	Yes	Yes
Was follow-up complete and if not were differences between groups in terms of their follow-up adequately described and analyzed?	Yes	Yes	Yes
Were participants analyzed in the groups to which they were	Yes	Yes	Yes

randomized?			
Were outcomes measured in the same way for treatment groups?	Yes	Yes	Yes
Were outcomes measured in a reliable way?	Yes	Yes	Yes
Was appropriate statistical analysis used?	Yes	Yes	Yes
Was the trial design appropriate and any deviations from the standard RCT design (individual randomization parallel groups) accounted for in the conduct and analysis of the trial?	Yes	Yes	Yes
Overall appraisal	Included	Included	Included

Supplementary Table S3. Quality Assessment of Cohort Studies					
Study	Meyer, SA, et al	Giuliano, M. et al (2017)	Giuliano, M. et al. (2023)	Kourtis, AP et al	Pirillo, MF et al
Were the two groups similar and recruited from the same population?	Yes	Not applicable	Yes	Yes	Not applicable
Were the exposures measured similarly to assign people to both exposed and unexposed groups?	Yes	Not applicable	Yes	Yes	Not applicable
Was the exposure measured in a valid and reliable way?	Yes	Yes	Yes	Yes	Yes
Were confounding factors identified?	Yes	Yes	No	No	Yes
Were strategies to deal with confounding factors stated?	Yes	No	No	No	No
Were the groups/participants free of the outcome at the start of the study (or at the moment of exposure)?	Yes	Unclear	Yes	No	Yes
Were the outcomes measured in a valid and reliable way?	Yes	Yes	Yes	Yes	Yes
Was the follow-up time	Yes	Yes	Yes	Yes	Yes

reported and sufficient to be long enough for outcomes to occur?					
Was follow-up complete, and if not, were the reasons for loss to follow-up described and explored?	Yes	No	No	No	No
Were strategies to address incomplete follow-up utilized?	Inapplicable	No	No	No	No
Was appropriate statistical analysis used?	Yes	Yes	Yes	Yes	Yes
Overall appraisal	Included	Included	Included	Included	Included

Supplementary Table S4. Quality Assessment of Case Reports

Study	AK Bhardwaj et al	Meyer et al.	Agarwal et al.	Bork et al.	Taddio et al.
Were patient's demographic characteristics clearly described?	Yes	Yes	Yes	Yes	Yes
Was the patient's history clearly described and presented as a timeline?	Yes	Yes	Yes	Yes	Yes
Was the current clinical condition of the patient on presentation clearly described?	Yes	Yes	Yes	Yes	Yes

Were diagnostic tests or assessment methods and the results clearly described?	Yes	Yes	Yes	Yes	Yes
Was the intervention(s) or treatment procedure(s) clearly described?	No	Yes	Yes	Yes	Yes
Was the post-intervention clinical condition clearly described?	No	Unclear	Yes	Yes	Unclear
Were adverse events (harms) or unanticipated events identified and described?	Yes	No	No	No	No
Does the case report provide takeaway lessons?	Yes	Yes	Yes	Yes	Yes
Overall appraisal	Not Included	Included	Included	Included	Included
